# Risk factors for childhood pneumonia at Adama Hospital Medical College, Adama, Ethiopia: a case-control study

**DOI:** 10.1186/s41479-022-00102-4

**Published:** 2022-12-05

**Authors:** Tsega-Ab Abebaw, William Kiros Aregay, Mulu Tugi Ashami

**Affiliations:** 1 Department of Public Health, GAMBY Medical and Business College, Addis Ababa, Ethiopia; 2 School of Medicine, Adama Hospital Medical College, Adama, Ethiopia

**Keywords:** Childhood pneumonia, Pneumonia, Risk factors, Under-five children, Adama, Ethiopia

## Abstract

**Background:**

Pneumonia is an acute respiratory infection of the lungs. A child dies of pneumonia every 39 s globally. Even though pneumonia affects children worldwide, the risk and repercussions of the disease are more prevalent in poor and middle-income nations. Despite the initiatives by the Ethiopian government, there are still numerous instances and deaths caused by childhood pneumonia. Therefore, this study aimed to identify the risk factors for pneumonia among 2–59 months-old children visiting Adama Hospital Medical College, Adama, Ethiopia.

**Methods:**

An institution-based unmatched case-control study was conducted among 124 cases and 124 controls from January 1, 2021, to March 15, 2021. Cases were selected using a consecutive sampling technique. For each case, the next patient from the same pediatric outpatient room who met the inclusion criteria was taken as a control. Data were collected using a pretested, structured interviewer-administered questionnaire containing sociodemographic, environmental, and nutritional factors, comorbid illnesses, and related care practices. A multiple logistic regression model was fitted.

**Results:**

Family size of ≥ 5 compared to < 5 (Adjusted odds ratio (AOR): 3.08, 95% CI: 1.23, 7.71), household monthly income of < 2500 compared to > 5000 birr (AOR: 3.94, 95% CI: 1.06, 14.6), use of charcoal as the main fuel for cooking (AOR: 7.03, 95% CI: 2.38, 20.78), and wood or dung as the main fuel for cooking compared to electricity (AOR: 6.58, 95% CI: 2.07, 20.9), malnutrition compared to no malnutrition (AOR: 4.77, 95% CI: 1.89, 12.06), diarrhea compared to no diarrhea in the past 2 weeks (AOR: 3.3, 95% CI: 1.52, 7.14) and upper respiratory tract infection (URTI) compared to no infection in the past 2 weeks (AOR: 3.29, 95% CI: 1.31, 8.23) were found to be risk factors for pneumonia.

**Conclusion:**

In this study, risk factors for pneumonia were family size, monthly income, type of energy used for cooking, malnutrition, and diarrhea or URTI in the past 2 weeks. Relatively simple interventions such as cooking with electricity, and other interventions like prevention, early detection and treatment of malnutrition, diarrhea, and URTI, and promotion of family planning are important.

## Background

Pneumonia is an acute respiratory infection of the lungs and the single largest infectious cause of death in children worldwide. A child dies of pneumonia every 39 s. It takes the lives of over 800,000 children under five years annually, or around 2,200 every day, contributing to 15% of all deaths of children under five years [1].

Even though pneumonia affects children worldwide, the risk and repercussions of the disease are more prevalent in poor and middle-income nations, where the bulk of deaths and cases occur [2]. Globally, there are over 1,400 cases of pneumonia per 100,000 children, or one case per 71 children every year, with the highest incidence occurring in South Asia (2,500 cases per 100,000 children) and West and Central Africa (1,620 cases per 100,000 children) [3].

The burden of pneumonia is disproportionately high in African children, with 36 million pneumonia cases annually [4]. In sub-Saharan Africa (SSA), the estimated proportion of deaths in children aged below five years attributed to pneumonia is 17–26% [5] and about 490,000 children under five years died of pneumonia in 2016 [6].

Ethiopia is one of the SSA countries with the highest rates of pneumonia. The first major cause of under-five mortality in the country is acute respiratory infection (18%) [7] and pneumonia contributes to 16.4% of all deaths, more than diarrhea, malaria, and acquired immunodeficiency syndrome (AIDS) and measles combined [8].

Children whose immune systems are compromised are at higher risk of developing pneumonia. Symptomatic human immunodeficiency virus (HIV) infection, measles, malnutrition, indoor air pollution, living in crowded homes, and parental smoking are among the globally known factors increasing a child’s risk of contracting pneumonia [9].

The Ethiopian government plans to reduce the under-five mortality rate to 31 and 14 per 1000 live births in 2025 and 2035, respectively, as part of the national goal of the health sector Transformation plan, which was launched in 2015 [10]. Despite the programs and initiatives, there are still numerous instances and deaths caused by childhood pneumonia.

There are some studies conducted regarding the predictors of childhood pneumonia in Ethiopia. The country is characterized by a great diversity of population and epidemiological situations. Risk factors may likely vary from one region to another. Therefore, it is necessary to investigate the specific situation of the study area to set up interventions appropriate to the local context. Hence, this study aimed to identify the risk factors for pneumonia among 2–59 months old children visiting the pediatric outpatient department (OPD) of Adama Hospital Medical College (AHMC).

## Methods

### Study design, area, and period

An institution-based unmatched case-control study was conducted from January 1, 2021, to March 15, 2021.

The study was conducted at AHMC, Adama, Ethiopia. Adama is a city located in the Oromia region, 100 km southeast of Addis Ababa, in the Great Rift Valley of East Africa. It is one of the major cities in Ethiopia, having an area of 29.86 square kilometers and a population of more than half a million. The city has four hospitals, eight public health centers, and more than one hundred thirty private health care facilities. AHMC is the first and only public and referral hospital in the city. As reported by the administrative office of AHMC, the hospital serves a population of more than 6 million from five regions (Oromia, Amhara, Afar, Somali, and Dire-Dawa). The hospital has a capacity of 527 beds for admission and serves an average of 1000 patients per day in 15 OPDs in 10 different specialties. The Department of Pediatrics is one of the treatment teams with the capacity to handle 40 cases per day at the Pediatric OPD.

### Population

The source population of the study were all 2–59 months old children visiting AHMC pediatric OPD for different reasons during the study period.

#### Cases

Cases were defined as 2–59 months old children who visited the Pediatric OPD of AHMC and were diagnosed with pneumonia within the study period. The assigned physician were the ones to make the diagnosis using the World Health Organization (WHO) Integrated Management of Childhood Illness (IMCI) guideline adopted by the Ethiopian Government.

#### Controls

Controls were defined as 2–59 months old children who visited the Pediatric OPD of AHMC and were diagnosed with non-pneumonia cases within the study period.

### Sample size determination

Sample size calculation for two population proportions was employed using EPI info version 7.2.4.0 software. The assumption was based on a 95% confidence level, 90% power, 1:1 case-to-control ratio, history of having no separate kitchen as the main predictor of Pneumonia, the percent of controls exposed as 3.4%, and the Odds Ratio (OR) of 5.37 from a case-control study in Northeast Ethiopia [11]. The estimated sample size was 236. After adding a 5% nonresponse rate, the total sample size becomes 248 (124 cases and 124 controls).

### Sampling procedure

Cases were selected using a consecutive sampling technique until the required sample size was attained. For each case of pneumonia, the next visitor or patient from the same pediatric OPD who met the inclusion criteria was taken as a control. Finally, mothers or caregivers of the cases and controls were interviewed.

### Inclusion and exclusion criteria

Mothers/caretakers who had children 2–59 months of age coming to AHMC for health care services were included in the study, and respondents who were critically ill during the data collection period were excluded from the study.

### Variables

#### Dependent variable

- Pneumonia.

#### Independent variables

- Sociodemographic variables (age, sex, birth order, family size, area of residence, age of mother, occupation of parents, education status of parents, marital status of parents, and average monthly income).
- Environmental factors (mainly used room for cooking, the place where the child stays during cooking, type of fuel used for cooking, presence of windows, source of water, hand washing before child feeding, cigarette smoking in the house, and domestic animals in the house).
- Nutrition and vaccination status (exclusive breastfeeding up to 6 months, current breastfeeding status, age when complementary feeding started, zinc supplementation, and malnutrition).
- Self or family history of illness (HIV status of the child, history of diabetes, diarrhea in the past two weeks, upper respiratory tract infection (URTI) in the past two weeks, pneumonia in the family in the past two weeks, history of measles illness, history of parental bronchial asthma).

### Operational definitions

#### Pneumonia

A child with cough or difficult breathing who has fast breathing and no general danger signs, no chest indrawing and no stridor when calm, was considered to have pneumonia.

#### Malnutrition

Malnutrition was considered when children had a mid-upper arm circumference (MUAC) < 125 mm or weight for length/height < -2 z-score (WHO standard).

#### Vaccination status

Children who had an age-inappropriate vaccination according to the Expanded Program on Immunization schedule, and those who had never been vaccinated were considered to have inadequate vaccination status. At the same time, those who had an age-appropriate vaccination and those who had completed vaccination were considered to have adequate vaccination status.

### Data collection procedure

The data were collected using a pretested, structured interviewer-administered questionnaire. The questionnaire included sociodemographic characteristics, environmental factors, nutritional factors, comorbid illnesses, and related care practices. The questionnaire was adapted from previous studies with minimal modification. After each interview, a record review was done to collect information on the MUAC, height, weight, and HIV status of the child.

### Data quality control

To assure the quality of data, the questionnaire was pretested with 5% of the targeted sample size at AHMC before running the actual data. Necessary modifications of the questionnaire were carried out based on the pretest feedback. The reliability of the questionnaires was checked. The data were collected under regular supervision after giving a one-day training for three data collectors and a supervisor before the initiation of the data collection process. The data collectors and the supervisor were nurses with prior training in IMCI and not an employee of AHMC. All questionnaires were reviewed and checked for completeness by the supervisors and principal investigator daily and the necessary feedback was being provided to data collectors.

### Data processing and analysis

The data were checked for completeness and consistency, and then cleaned and entered using Epi info version 7.2 0.4.0 and exported to SPSS version 26 for analysis. The descriptive analyses were presented by frequency tables and percentages. A multiple logistic regression model was fitted to find factors associated with pneumonia. Initially, all variables were screened by carrying out binary logistic regression analyses. Then, variables that had a *p*-value of less than 0.2 were taken into the multivariable logistic regression model to identify confounders and the independent effect of different factors on the occurrence of pneumonia. A *p*-value less than 0.05 and an adjusted odds ratio with 95% CI were used to determine the presence and degree of association.

## Results

### Socio-demographic characteristics

A total of 237 study participants (118 cases and 119 controls) enrolled in the study. The response rate of cases was 95.2% and controls 96%. Of these enrolled, 70 (59.3%) cases and 69 (58%) controls were male. The mean (± SD) age of the children was 19.2 (± 16.55) months and 20.9 (± 17.72) months for cases and controls, respectively. Urban residents accounted for 56 (47.5%) of cases and 82 (68.9%) of controls. Most of the mothers of case 113 (95.8%) and control 111 (93.3%) were married. In most of the cases, 66 (55.9%) had a household monthly income of 2500–5000 birr while 55 (46.2%) of the controls had < 2500 birr (Table 1).

**Table 1:** Socio-demographic, environmental, nutritional, and disease-related characteristics of 2–59 months old children. Adama, Ethiopia, 2021

Variables	Pneumonia status	
	Cases, n (%)	Controls, n (%)
**Age**		
2–11 months	52 (44.1)	49 (41.2)
12–35 months	33 (28.0)	32 (26.9)
36–59 months	33 (28.0)	38 (31.9)
**Sex**		
Male	70 (59.3)	69 (58.0)
Female	48 (40.7)	50 (42.0)
**Birth order**		
1st	26 (22.0)	22 (18.5)
2nd	48 (40.7)	46 (38.6)
3rd and above	44 (37.3)	41 (34.4)
**Family size**		
< 5	12 (10.2)	43 (36.1)
≥ 5	106 (89.8)	76 (63.9)
**Area of residence**		
Urban	56 (47.5)	82 (68.9)
Rural	62 (52.5)	37 (31.1)
**Age of mother**		
18–24 years	50 (42.4)	49 (41.2)
25–34 years	40 (33.9)	43 (36.1)
≥ 35 years	28 (23.7)	27 (22.7)
**Marital status of mother**		
Married	113 (95.8)	111 (93.3)
Unmarried	5 (4.2)	8 (6.7)
**Education status of mother**		
No formal education	63 (53.4)	57 (47.9)
Primary	41 (34.7)	47 (39.5)
Secondary and above	14 (11.9)	15 (12.6)
**Education status of father**		
No formal education	64 (54.2)	28 (23.5)
Primary	15 (12.7)	16 (13.4)
Secondary and above	39 (33.1)	75 (63.0)
**Mother’s occupation**		
Housewife	88 (74.6)	80 (67.2)
Civil servant	16 (13.6)	19 (16.0)
Merchant	10 (6.8)	13 (10.9)
Others	4 (5.1)	7 (5.9)
**Father’s occupation**		
Farmer	46 (39.0)	40 (33.6)
Civil servant	26 (22.0)	32 (26.9)
Merchant	14 (11.9)	18 (15.1)
Daily laborer	24 (20.3)	20 (16.8)
Others	8 (6.8)	9 (7.6)
**Household monthly income**		
< 2500 birr	45 (38.1)	55 (46.2)
2500–5000 birr	66 (55.9)	39 (32.8)
> 5000 birr	7 (5.9)	25 (21.0)
**Mainly used room for cooking**		
Separate kitchen	69 (58.5)	91 (76.5)
Living room	49 (41.5)	28 (23.5)
**Child stay during cooking**		
Inside cooking room	26 (22.0)	24 (22.0)
Outside cooking room	92 (78.0)	95 (78.0)
**Presence of window**		
Yes	65 (55.1)	79 (66.4)
No	53 (44.9)	40 (33.6)
**Source of water**		
Pipe water	115 (97.5)	114 (95.8)
Others	3 (2.5)	5 (4.2)
**Hand washing before child feeding**		
Only water	39 (33.1)	34 (28.6)
Water and soap	79 (66.9)	85 (71.4)
**Cigarette smoking in the house**		
Yes	13 (11.0)	18 (15.1)
No	105 (89.0)	101 (84.9)
**Mostly used fuel for cooking**		
Electricity	17 (14.4)	34 (28.6)
Stove or gasoil	37 (31.4)	48 (40.3)
Charcoal	34 (28.8)	24 (20.2)
Wood or dung	30 (25.4)	13 (10.9)
**Domestic animals in the house**		
Yes	26 (22.0)	22 (18.5)
No	92 (78.0)	97 (81.5)
**Breast feeding up to 6 months**		
Exclusive	94 (79.7)	108 (90.8)
Non-exclusive	24 (20.3)	11 (9.2)
**Current breast-feeding status**		
Yes	69 (58.5)	65 (54.6)
No	49 (41.5)	54 (45.4)
**Age when complementary feeding started**		
Before 6 months	24 (20.3)	11 (9.2)
After 6 months	75 (63.6)	85 (71.4)
Not started	19 (16.1)	23 (19.3
**Zinc supplementation**		
Yes	20 (16.9)	22 (18.5)
No	98 (83.1)	97 (81.5)
**Malnutrition**		
Yes	52 (44.1)	14 (11.8)
No	66 (55.9)	105 (88.2)
**Vaccination status**		
Adequate	87 (73.7)	104 (87.4)
Inadequate	31 (26.3)	15 (12.6)
**HIV status**		
Negative	108 (91.5)	56 (47.1)
Positive	10 (8.5)	0 (0.0)
Not determined	0 (0.0)	63 (52.9)
**Diabetic**		
Yes	1 (0.8)	0 (0.0)
No	117 (99.2)	119 (100.0)
**Diarrhea in the past 2 weeks**		
Yes	54 (45.8)	27 (22.7)
No	64 (54.2)	92 (77.3)
**URTI in the past 2 weeks**		
Yes	99 (83.9)	70 (58.8)
No	19 (16.1)	49 (41.2)
**Pneumonia in the family in the past 2 weeks**		
Yes	2 (1.7)	0 (0.0)
No	116 (98.3)	119 (100.0)
**History of measles illness**		
Yes	2 (1.7)	1 (0.8)
No	116 (98.3)	118 (99.2)
**History of Parental Asthma**		
Yes	6 (5.1)	5 (4.2)
No	112 (94.9)	114 (95.8)

### Environmental characteristics

Forty-nine (41.5%) of the cases and 22 (23.5%) of the controls used their living room as the main room for cooking. An equal proportion (78%) of the cases and controls stayed outside the cooking room while cooking. Using stove or gasoil for cooking accounted for 37 (31.4%) cases and 48 (40.3%) controls. Handwashing has been in practice with water and soap before feeding the child in 79 (66.9%) of the cases and 85 (71.4%) controls (Table 1).

### Nutrition and vaccination status

Among the respondents, 94 (79.7) cases and 108 (90.8) controls had a history of exclusive breastfeeding. Malnutrition was present in 52 (44.1%) cases, whereas 105 (88.2%) controls were free from it. Adequate vaccination status accounted for 87 (73.7%) cases and 104 (87.4%) of controls (Table 1).

### Self or family history of illness

Regarding the HIV status of the respondents, 108 (91.5%) cases tested negative, and 10 (8.5%) tested positive. Fifty-six (47.1%) of the controls tested negative, and the status of 63 (52.9%) controls was not determined. Fifty-four (45.8%) cases had a history of diarrhea in the past two weeks, while only 27 (22.7%) of the controls had a history of diarrhea. The history of URTI in the past two weeks accounts for 99 (83.9%) of cases and 70 (58.8%) of controls (Table 1).

### Risk factors for childhood pneumonia

A binary logistic regression model was applied to see the association between the dependent variable and every independent variable. According to the finding, 11 variables (family size, area of residence, education status of the father, household income, mainly used room for cooking, mostly used fuel for cooking, breastfeeding for up to 6 months, presence of malnutrition, diarrhea in the past two weeks, URTI in the past two weeks and vaccination status) had an association with pneumonia. A multivariable logistic regression analysis was employed to look for confounding factors. According to this analysis, 4 of the above variables (mainly room for cooking, area of residence, breastfeeding up to 6 months, and vaccination status) lost their association.

Study participants with a family size of ≥ 5 were three times (AOR: 3.08, 95% CI: 1.23, 7.71) more likely to have pneumonia than those who had < 5. Children of fathers with no formal education were about five times (AOR: 4.88, 95% CI: 1.77, 13.46) more likely to have had pneumonia than those with secondary and above education. Respondents with a household monthly income of < 2500 birr were about four times (AOR: 3.94, 95% CI: 1.06, 14.6) more likely to have pneumonia than those who had > 5000 birr.

Mostly used fuel for cooking is one of the variables which had a significant association with the dependent variable. Those who used charcoal and wood or dung were about seven times (AOR: 7.03, 95% CI: 2.38, 20.78) and more than six times (AOR: 6.58, 95% CI: 2.07, 20.9) more likely to have had pneumonia than those who use electricity respectively.

Children who had malnutrition were more than five times (AOR: 4.77, 95% CI: 1.89, 12.06) and those who had diarrhea and URTI in the past two weeks were more than three times (AOR: 3.3, 95% CI: 1.52, 7.14) and (AOR: 3.29, 95% CI: 1.31, 8.23) more likely to had pneumonia than those who had no malnutrition and diarrhea and URTI in the past two weeks respectively (Table 2).

**Table 2:** Factors associated with pneumonia among 2–59 months old children in Adama, Ethiopia, 2021

Variables	Pneumonia status		Odds ratio (OR) at 95% CI	
	Cases, n (%)	Controls, n (%)	Crude OR	Adjusted OR
**Family size**				
< 5	12 (10.2)	43 (36.1)	1	1
≥ 5	106 (89.8)	76 (63.9)	**5 (2.47, 10.11)**	**3.08 (1.23, 7.71)**
**Area of residence**				
Urban	56 (47.5)	82 (68.9)	1	1
Rural	62 (52.5)	37 (31.1)	**2.45 (1.44, 4.17)**	0.58 (0.2, 1.68)
**Education status of father**				
No formal education	64 (54.2)	28 (23.5)	**4.4 (2.44, 7.92)**	**4.88 (1.77, 13.46)**
Primary	15 (12.7)	16 (13.4)	1.8 (0.81, 4.03)	1.56 (0.47, 5.19)
Secondary and above	39 (33.1)	75 (63.0)	1	1
**Household monthly income**				
< 2500 birr	45 (38.1)	55 (46.2)	**2.92 (1.16, 7.38)**	**3.94 (1.06, 14.6)**
2500–5000 birr	66 (55.9)	39 (32.8)	**6.04 (2.39, 15.27)**	2.14 (0.44, 10.4)
> 5000 birr	7 (5.9)	25 (21.0)	1	1
**Mainly used room for cooking**				
Separate kitchen	69 (58.5)	91 (76.5)	1	1
Living room	49 (41.5)	28 (23.5)	**2.31 (1.32, 4.04)**	1.09 (0.49, 2.44)
**Presence of window in the house**				
Yes	65 (55.1)	79 (66.4)	1	1
No	53 (44.9)	40 (33.6)	1.61 (0.95, 2.72)	1.57 (0.76, 3.27)
**Mostly used fuel for cooking**				
Electricity	17 (14.4)	34 (28.6)	1	1
Stove or gasoil	37 (31.4)	48 (40.3)	1.54 (0.75, 3.18)	1.89 (0.73, 4.9)
Charcoal	34 (28.8)	24 (20.2)	**2.83 (1.3, 6.2)**	**7.03 (2.38, 20.78)**
Wood or dung	30 (25.4)	13 (10.9)	**4.62 (1.93, 11.05)**	**6.58 (2.07, 20.9)**
**Breast feeding up to 6 months**				
Exclusive	94 (79.7)	108 (90.8)	1	1
Non-exclusive	24 (20.3)	11 (9.2)	**2.51 (1.17, 5.39)**	0.9 (0.32, 2.52)
**Presence of malnutrition**				
Yes	52 (44.1)	14 (11.8)	**5.11 (3.04, 11.5)**	**4.77 (1.89, 12.06)**
No	66 (55.9)	105 (88.2)	1	1
**Diarrhea in the past 2 weeks**				
Yes	54 (45.8)	27 (22.7)	**2.88 (1.64, 5.04)**	**3.3 (1.52, 7.14)**
No	64 (54.2)	92 (77.3)	1	1
**URTI in the past 2 weeks**				
Yes	99 (83.9)	70 (58.8)	**3.65 (1.98, 6.73)**	**3.29 (1.31, 8.23)**
No	19 (16.1)	49 (41.2)	1	1
**Vaccination status**				
Adequate	87 (73.7)	104 (87.4)	1	1
Inadequate	31 (26.3)	15 (12.6)	**2.47 (1.25, 4.87)**	1.85 (0.75, 4.56)

NB Crude OR and Adjusted OR written in bold indicates statistical significance or *P value < 0.05*

## Discussion

The study revealed that, among those who visited the pediatric OPD, children who live in families with five or more members had a triple risk of presentation with pneumonia than those with a family size of less than five. Crowding appeared to increase the risk of childhood pneumonia in a study conducted in Brazil [12], and having three or more children at home showed statistical significance in research conducted in the Netherlands [13]. Studies conducted in Ethiopia [14, 15] also showed similar results. The reasonably increased risk of respiratory infection by increasing the opportunity for cross-infection among the family could explain the case.

The study also showed that, among those who visited the pediatric OPD, children of fathers with no formal education had five times increased risk of presentation with pneumonia than those who had secondary and above education status. However, mothers’ education status showed no significance in our study. Consistent with our study, many other studies from the same country showed no significant association between maternal education and childhood pneumonia [11, 14–20]. Similar to our study, a higher father’s literacy level resulted in a diminished risk of pneumonia in a study conducted in the Amhara Region [16]. The reason for these associations could be that fathers have a vital and strategic role in the hierarchy of a family. Their role in making decisions may greatly determine the foundation of a family, including maintaining health and vaccination of children. The consistent findings regarding the lack of association between a mother’s education and childhood pneumonia may need further investigation.

In our study, household monthly income was also one of the variables significantly associated with pneumonia among children who visited the pediatric OPD. Those whose families earn < 2500 birr had four times the risk of presentation with pneumonia than those who get > 5000 birr. Other studies in India [21–23], Brazil [24], Zambia [25], and Ethiopia [17] also confirmed that low socio-economic class is a risk factor. The impact of economic conditions on health status has long been recognized. Low income may lead to poor nutrition, poor housing, and little or no preventive medical care like immunization which may greatly contribute to acquiring communicable diseases.

Breathing clean air at home is essential for children’s healthy development, but widespread dependence on solid fuels and kerosene for cooking results in far too many children living in heavily polluted home environments [26]. Our study revealed that, among those who visited the pediatric OPD, those whose families use charcoal and wood or dung for cooking had about seven times and more than six times the risk of presentation with pneumonia than those who use electricity. Most existing reports consistently indicate that indoor air pollution from biomass fuels is indeed a risk factor for pneumonia. Studies conducted in Indonesia [27], Burkina Faso [28], Kenya [29], and Ethiopia [17] support the finding of this study. Air pollutants associated with biomass fuel may adversely affect specific and nonspecific host defenses of the respiratory tract against pathogens.

Our study revealed that, among those who visited the pediatric OPD, children with malnutrition had more than five times the risk of presentation with pneumonia than those who had no malnutrition. It is consistent with results from India [21], Brazil [12], Indonesia [27], and Ethiopia [14, 18]. The possible explanation can be the effect of malnutrition to elicit dysfunction in the immune system and promote increased vulnerability of the children to infection. From those who visited the pediatric OPD, children who had diarrhea and URTI in the past two weeks before our study were more than three times highly likely to have had pneumonia than those who hadn’t. Similar to this study, a report from Northeast Ethiopia [11] and Amhara Region, Ethiopia [16, 19], showed children who had diarrhea had a higher risk of presentation with pneumonia. Studies also showed a clear association between the occurrence of pneumonia and previous URTIs [11, 13, 14, 19, 20]. Higher infection susceptibility in these children could be a possible mechanism causing predisposition to pneumonia. A concomitant illness like diarrhea may also lower immunity, making children more susceptible to diseases like pneumonia.

### Limitations

The institution-based nature of the study could limit the generalizability of the findings. Other limitations of the study could be a recall bias and the diagnosis of pneumonia which was based on the clinical IMCI classification guideline, which could introduce misclassification bias.

## Conclusion

Family size, education status of the father, household monthly income, mostly used fuel for cooking, malnutrition, and history of diarrhea, and URTI in the past two weeks was found to be the risk factors for pneumonia. Relatively simple interventions such as cooking with electricity, and other interventions like prevention, early detection and treatment of malnutrition, diarrhea, and URTI, and promotion of family planning are important. Prevention of the risk factors should get priority over treatment of cases, and health care facilities should work with other stakeholders to reverse these risk factors.

## Data Availability

The datasets used and/or analyzed during the current study are available from the corresponding author on reasonable request.

## Abbreviations

AHMC: Adama Hospital Medical College
AIDS: Acquired Immunodeficiency Syndrome
AOR: Adjusted Odds Ratio
COR: Crude Odds Ratio
HIV: Human Immunodeficiency Virus
IMCI: Integrated Management of Childhood Illness
MUAC: Mid-Upper Arm Circumference
OPD: Out Patient Department
OR: Odds Ratio
SSA: Sub-Saharan Africa
URTI: Upper Respiratory Tract Infection
WHO: World Health Organization
